# THE UNIQUE CHARACTERISTICS OF “FAMILIES OF CHOICE” AND BIOLOGICAL FAMILIES OF LGBT ADULTS

**DOI:** 10.1093/geroni/igad104.3667

**Published:** 2023-12-21

**Authors:** Ella Cohn-Schwartz, Sigal Gooldin, Yaacov Bachner

**Affiliations:** Ben Gurion University, Haifa, HaZafon, Israel; The Association for LGBTQ Equality, Tel Aviv, Tel Aviv, Israel; Ben-Gurion University of the Negev, Beer-Sheva, HaDarom, Israel

## Abstract

This study joins a growing body of research on the unique families of aging individuals in sexual minorities. We explore the “families of choice” (who are close enough to be considered as family) and biological families of LGBT adults and their associations with mental health. Data for this study was collected via an online survey with self-identified lesbian, gay, bi-sexual and transgender (LGBT) adults aged 50+ in Israel (n=483). The participants were asked about characteristics of the relationship with their families of choice and biological families and about experiencing depressive symptoms. Descriptive results indicated that most participants had a family of choice, numbering five people on average. These families of choice were mostly composed of partners and friends, but also of the family members of one’s partner, and of ex-partners and colleagues. They reported having about four close biological family members, mostly children, parents, siblings, and nieces. Several differences emerged when comparing the two types of families: The relationships with biological families were more stable and their biological family members were contacted more often. On the other hand, families of choice were more likely to accept their sexual orientation and the relationship with them had fewer negative aspects. Regression analyses showed that individuals had more depressive symptoms if the relationship with their families of choice was less stable and if there were more negative relationships with both types of families. These findings shed light on the unique sources of support among LGBT adults and their associations with mental health.

